# Modified balloon-assisted shoehorn technique for transcatheter aortic valve implantation in functionally horizontal anatomy: a case report

**DOI:** 10.1093/ehjcr/ytag616

**Published:** 2026-08-21

**Authors:** Nilkanth Patil, Rajeev Menon, Uday Kiran Anne, Pragathi Gurram, Anuj Kapadiya

**Affiliations:** Department of Cardiology, AIG Hospitals, Gachibowli, Hyderabad 500032, India; Department of Cardiology, AIG Hospitals, Gachibowli, Hyderabad 500032, India; Department of Cardiology, AIG Hospitals, Gachibowli, Hyderabad 500032, India; Department of Cardiology, AIG Hospitals, Gachibowli, Hyderabad 500032, India; Department of Cardiology, AIG Hospitals, Gachibowli, Hyderabad 500032, India

**Keywords:** TAVI, Horizontal anatomy, BAV, Difficult THV delivery

## Abstract

**Background:**

Transcatheter aortic valve implantation (TAVI) in bicuspid aortic valve (BAV) poses unique challenges. We report a case in which an apparently benign anatomy resulted in failure of conventional delivery strategies and required a novel balloon-assisted bailout technique.

**Case summary:**

A 59-year-old man with a background history of liver cirrhosis presented with severe aortic stenosis due to type 1 BAV. The patient was in Child-Pugh class A with model for end-stage liver disease (MELD) score of 14; hence, he was planned for TAVI in view of high surgical risk. Pre-procedural computed tomography showed a favourable left ventricular (LV)-aortic angle, but the fused non-coronary and right-coronary cusp behaved like functional horizontal anatomy. Various previously described wire and balloon-assisted techniques failed to deliver the valve. A novel balloon-assisted modified ‘shoe-horn’ technique, by inflating a 16 mm balloon in non-coronary sinus, altered the delivery trajectory and enabled successful valve delivery.

**Discussion:**

BAV with right non-coronary cusps fusion may behave like horizontal anatomy, despite favourable LV-aortic angle. A modified balloon-assisted shoe-horn technique can facilitate valve delivery when conventional methods fail. This case highlights an under-recognized mechanism of valve delivery failure in BAV and emphasizes the importance of preparedness for advanced bailout strategies.

Learning points

- BAV with RCC-NCC fusion may behave as functional horizontal anatomy, leading to unexpected THV delivery failure despite a favourable left ventricular-aortic angle.
- A modified shoe-horn technique with a fully inflated balloon within the non-coronary can facilitate valve delivery when conventional strategies fail.

## Introduction

Transcatheter aortic valve Implantation (TAVI) in bicuspid aortic valve (BAV) poses unique challenges.^1^ We report a case in which an apparently benign anatomy resulted in failure of conventional delivery strategies and required a novel balloon-assisted bailout technique.

## Summary figure

**Figure:**
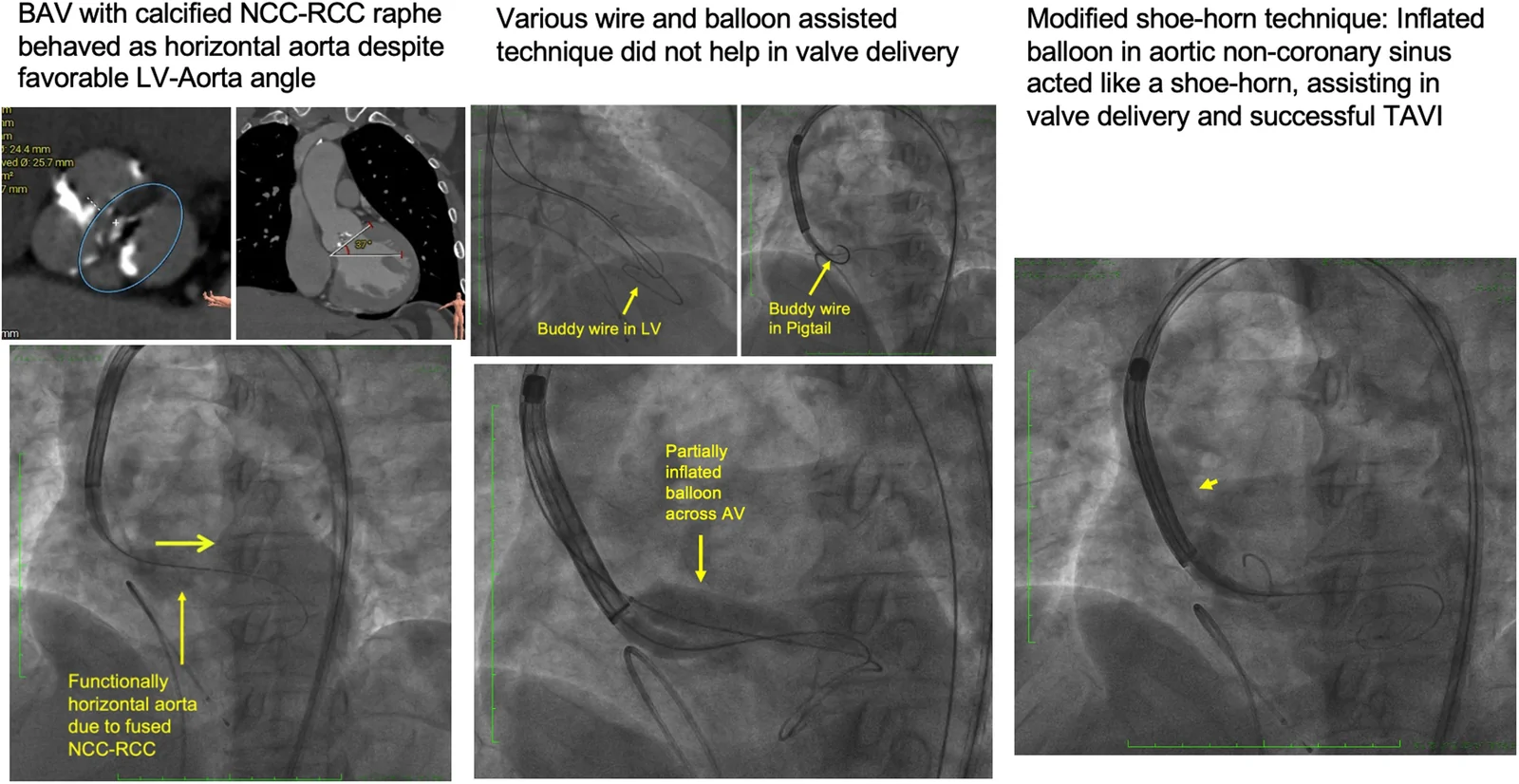


## Case presentation

A 59-year-old man presented with exertional dyspnoea of NYHA class II severity. He was a known case of compensated liver cirrhosis (Child–Pugh class A). Cardiovascular examination revealed an ejection systolic murmur of grade 3/6 severity in the aortic area. Transthoracic echocardiography showed severe aortic stenosis with peak and mean aortic gradients of 73 and 43 mmHg, respectively, an aortic valve area of 0.8 cm^2^; normal left ventricular (LV) function and other cardiac valves. The patient had underlying liver cirrhosis with portal hypertension and a prior episode of melena due to grade II oesophageal varices, treated with endoscopic variceal ligation.

## Investigations

His hepatic parameters revealed normal liver enzymes, serum bilirubin of 1.4 mg/dL, serum albumin of 2.8 mg/dL, an INR of 1.4 and serum creatinine of 1.3 mg/dL. He was in compensated live cirrhosis (Child–Pugh class A) with a MELD score of 14, indicating an intermediate risk profile. Following a multi-disciplinary discussion involving the Heart Team, hepatologist, and gastroenterologist, he was planned for TAVI in view of high surgical risk associated with cardiac surgery in liver cirrhosis.^2^

Pre-procedural computed tomography showed a type 1 BAV with fusion of non-coronary (NCC) and right coronary cusp (RCC) and a calcified raphe (*Figure 1B*). The annulus perimeter was 98.4 mm. The sinus anatomy, coronary heights and vascular access were suitable for a transfemoral TAVI (*Figure 1*). Left Ventricular—Aortic (LV-Aortic) angle was favourable at 37 degrees (*Figure 1C*). Based on annular dimensions, an Evolute-R 34 mm CoreValve (Medtronic, MN, USA) was chosen. The choice of self-expanding valve (SEV) was based on lower risk of annular rupture as compared to balloon-expandable valve (BEV) in heavily calcified anatomy and the institutional procedural familiarity with the SEV.

**Figure 1:**
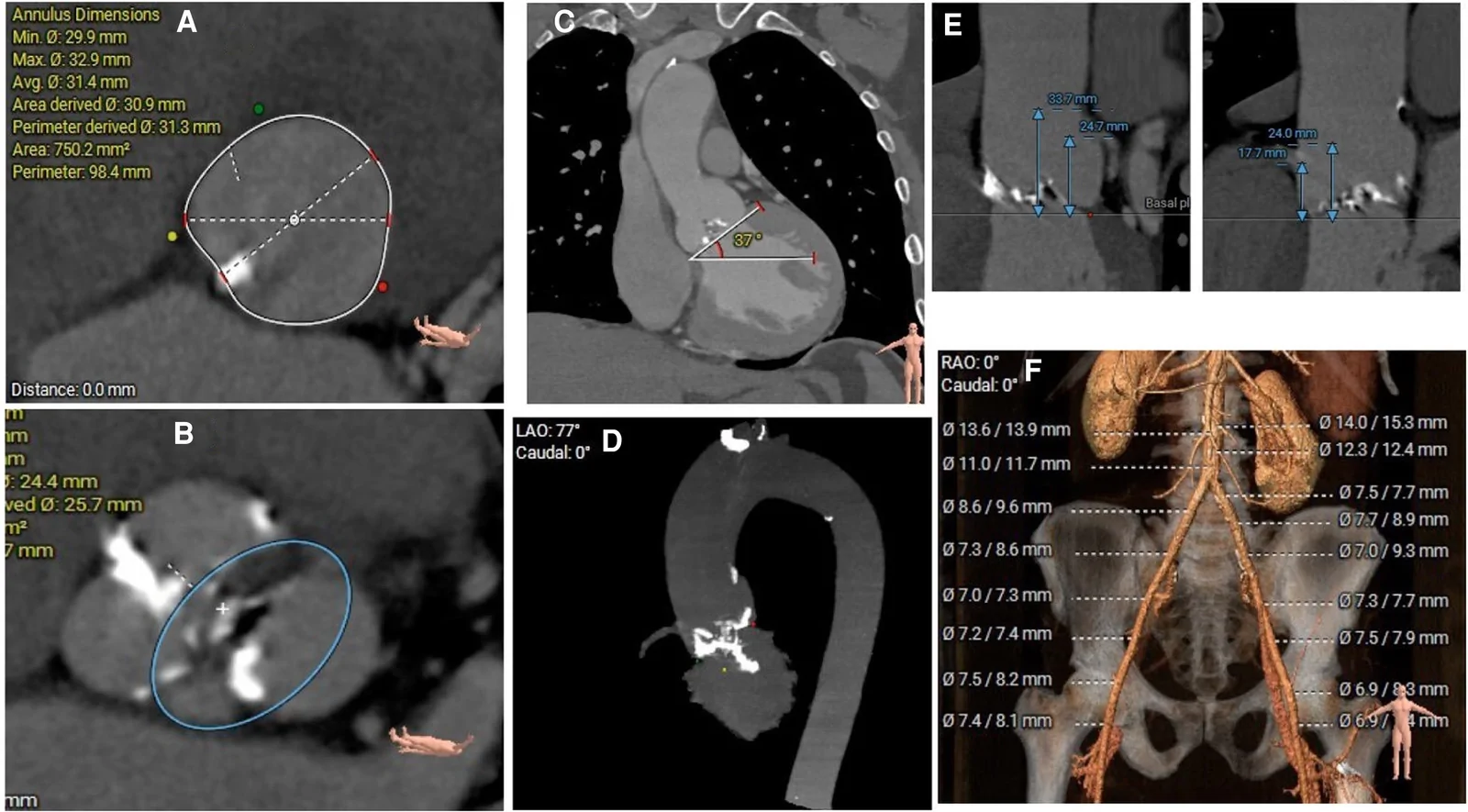
Computed tomography of AV; (*A*) aortic annulus with diameter of 31.4 mm and perimeter of 98.4 mm; (*B*) Type 1 Bicuspid aortic valve with non and right coronary cusp fusion with calcified raphe; (*C*) Favorable left ventricular aortic angle of 37 degrees; (*D*) Aortic calcification extending into LVOT; (*E*) Adequate coronary heights of 24.7 mm on left and 17.7 on right; (*F*) Peripheral vascular access adequate for transfemoral TAVI.

## Management

The procedure was done under conscious sedation. Right femoral artery was used as primary access and the left femoral artery as secondary access for pigtail placement in the non-coronary cusp. Left femoral vein access was used for a 5-F balloon-tipped temporary pacing catheter (BARD Medical). Aortic valve (AV) was crossed and Confida-Brecker guidewire (Medtronic, Minneapolis, MN, USA) was placed in LV. Baseline gradients were 45 mm Hg. AV was predilated with 23 mm NuCLEUS (Numed, Inc., Hopkinton, NY, USA) balloon (see Supplementary material online, *Video S1*).

An Evolute-R 34-mm transcatheter heart valve (THV) was then advanced; however, the transcatheter heart valve (THV) could not cross the AV. Due to the outer curvature bias and fused NCC and RCC, the anatomy behaved like a horizontal aorta, and the valve crossing was unsuccessful despite multiple pull–push manoeuvres (see Supplementary material online, *Video S2*, *Figure 2C*).

**Figure 2:**
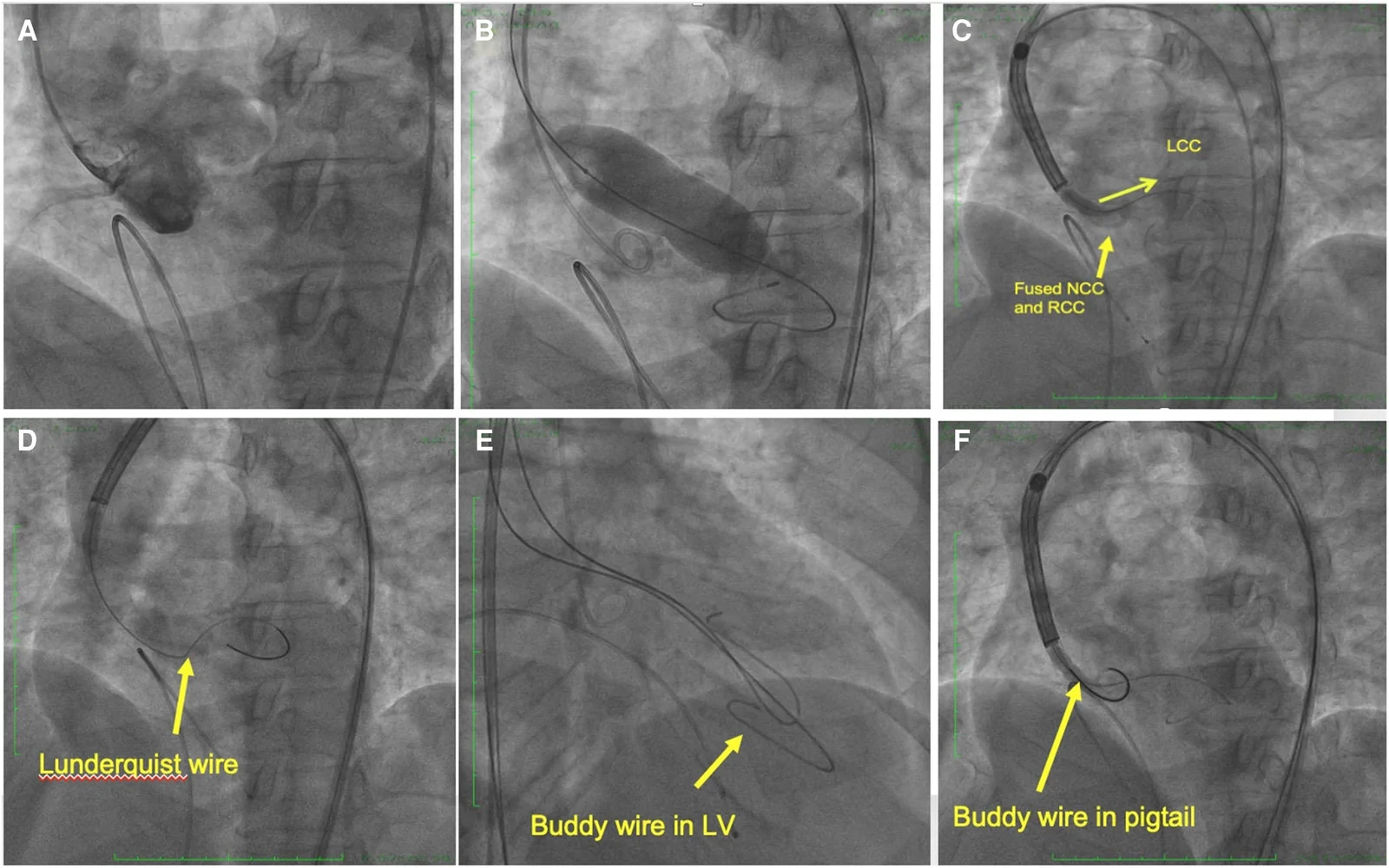
Pre-dilatation: (*A*) baseline aortic root angiogram; (*B*) pre-dilatation with 23 mm balloon; (*C*) calcified raphe between right-noncoronary cusp and outer curvature bias of aorta causing functionally horizontal anatomy; (*D–F*) wire assisted techniques: using a stiffer wire (lunderquist wire) (*D*) buddy wire in LV to straighten the LV-aortic angle and increase the support (*E*) buddy wire in pigtail in the non-coronary cusp to make LV-aortic angle favorable to cross (*F*).

Repeat pre-dilatation was performed with a larger 25 mm balloon (see Supplementary material online, *Video S3*), but the THV still failed to cross. To increase the support, Confida guidewire was exchanged for a Lunderquist extra-stiff guidewire (Cook Medical, Bloomington, IN, USA); however, this also did not facilitate valve crossing. Additional buddy wire techniques were attempted, including placement of two guidewires in the LV and positioning of the Lunderquist wire within the pigtail catheter in NCC. Both strategies were unsuccessful (see Supplementary material online, *Video S4*, *Figure 2D–2F*).

Different combinations of balloon-assisted technique were then attempted, including advancing the THV during partial inflation and while deflating the 16 mm Z-MED (B. Braun Interventional System, Bethlehem, PA, USA) across AV; however, these approaches failed (*Figure 3*, Supplementary material online, *Video S5*). Once the described methods did not succeed, we positioned a large 16 mm Z-MED balloon in NCC, inflated the balloon, and then were able to successfully cross the AVby THV sliding over the inflated balloon (*Figure 4B*, Supplementary material online, *Video S6*). Valve was deployed in standard fashion under controlled pacing. There was mild frame under-expansion which was corrected by post-dilatation with a 25 mm NuCLEUS balloon (*Figure 5*, Supplementary material online, *Video S7*). Final aortic root angiogram showed mild paravalvular leak with minimal gradients of 2–4 mmHg.

**Figure 3:**
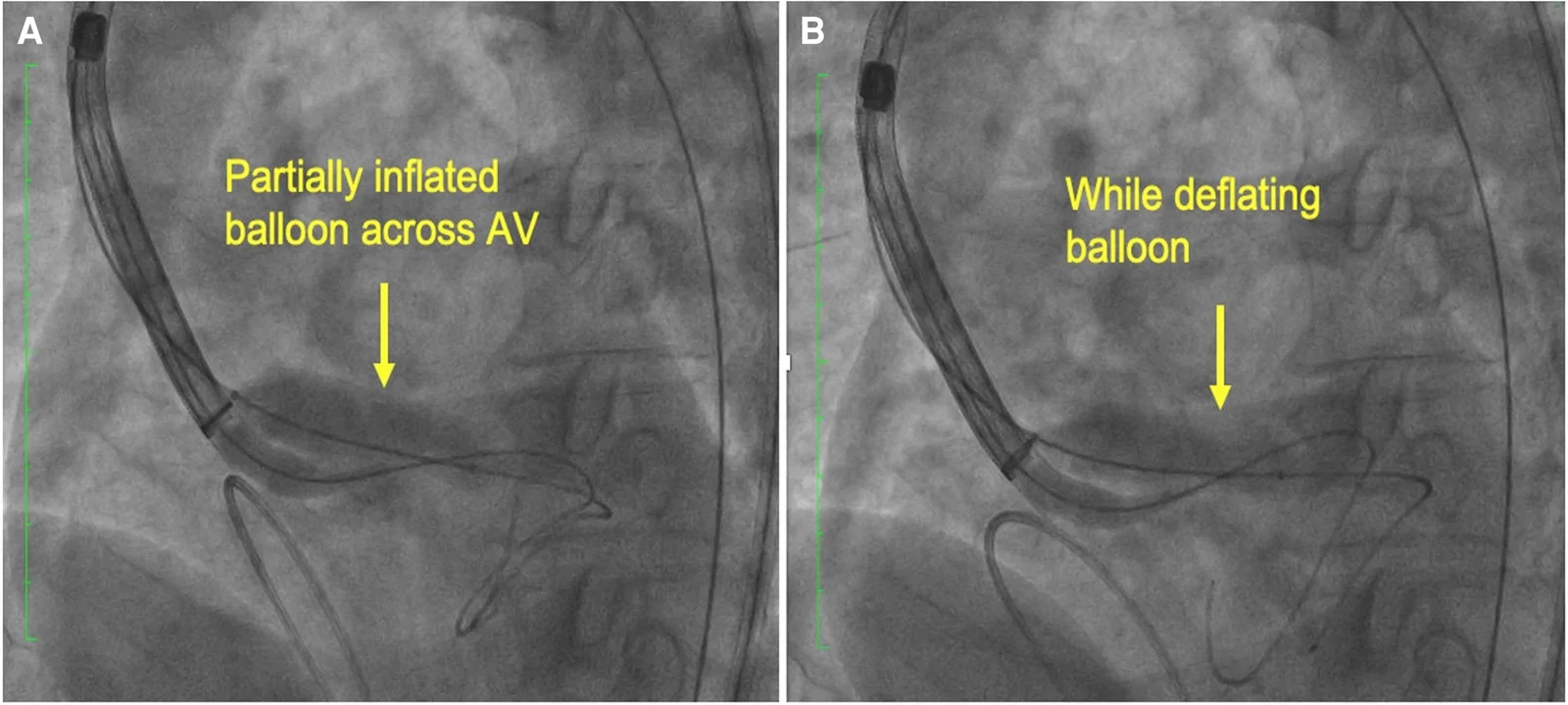
Balloon-assisted techniques: a 16 mm balloon inflated across aortic valve and the THV was attempted to deliver during balloon inflation (*A*) and while deflating (*B*) (shoehorn technique).

**Figure 4:**
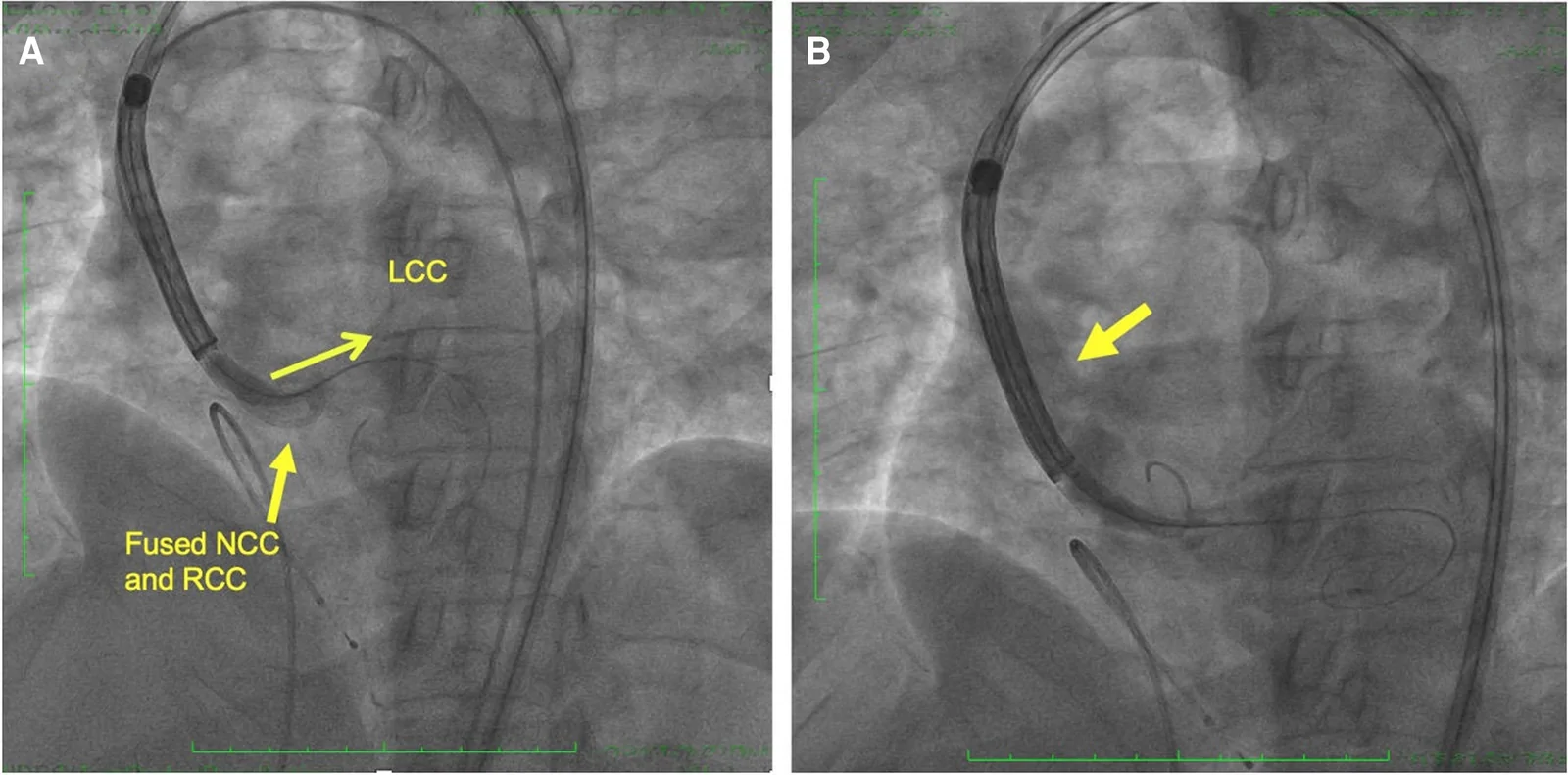
(*A*) Fusion of non-coronary and right-coronary cusps behaving like horizontal anatomy. (*B*) Modified shoe-horn technique: A 16 mm Z-Med balloon fully inflated in NCC of aorta (yellow arrow) acted as modified shoe-horn, which assisted the valve crossing.

**Figure 5:**
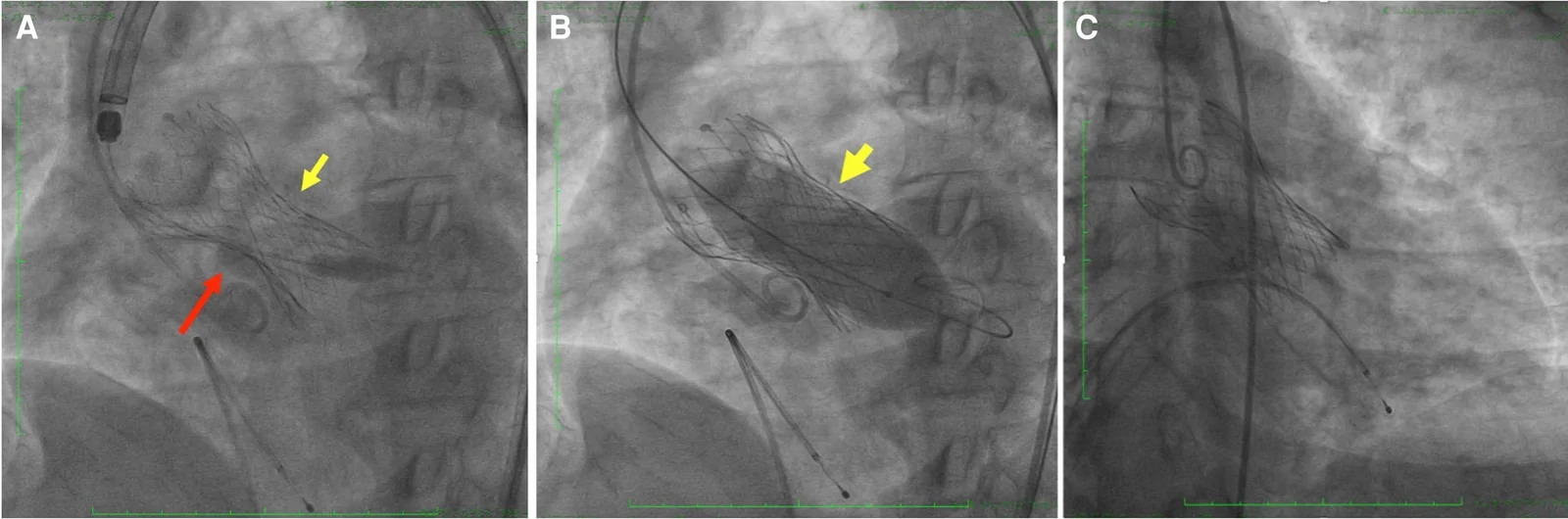
Final result: (*A*) valve deployment with under-expanded frame (yellow arrow) due to calcium towards non-coronary cusp (red arrow); (*B*) post dilatation with 25 mm balloon causing expansion of valve frame (yellow arrow), valve mainly expands away from the calcified raphe (and towards LCC); (*C*) final aortic root angiogram showing fully expanded valve, no PVL or other complications.

## Outcome and follow-up

Patient remained haemodynamically stable throughout the post-procedure period and was discharged on third post-procedure day without any complications. Patient was asymptomatic from the cardiac point of view at more than 1 year of follow-up.

## Discussion

TAVI in BAV has been associated with higher rates of complications and increased procedural complexity.^1,3^ This is due to heterogeneous leaflet morphology, bulky, asymmetrical calcifications (often involving raphe), elliptical annular geometry, and associated aortopathy.^1,3^ Current AHA/ACC and ESC guidelines recommend surgery as the first-line treatment for severe aortic stenosis in BAV, particularly in relatively young patients.^4,5^

Although the patient was 59 years old chronologically, the Heart Team’s decision-making was guided by biological risk and expected survival rather than age alone. The patient had cirrhosis with prior decompensation, portal hypertension with variceal bleeding, and overall fragility, placing him at increased risk of perioperative complications. Conventional surgical risk scores (STS-PROM and EuroSCORE II) were not markedly elevated. However, these models do not fully account for the impact of liver cirrhosis, which is an independent predictor of adverse surgical outcomes.^6,7^ While BEV may offer advantages in younger patients with respect to future coronary access and re-intervention, In this case, SEV was selected based on institutional experience and prioritization of procedural safety, as competing mortality risks outweighed long-term device related considerations.

A pre-procedural CT plays a crucial role in anticipating THV delivery challenges. A steep LV-Aortic angle predicts difficult THV delivery, especially for SE valves.^8^ An angle >48 degrees predicts difficult valve delivery and an angle of >70 degrees necessitates advanced delivery methods.^8^ In our case, the LV-Aortic angle was favourable (37 degrees), hence the difficulty to deliver the valve was not anticipated. The combination of THV trajectory along the outer curvature of the aorta and raphe between RCC and NCC made it behave like horizontal anatomy (*Figure 3*, Supplementary material online, *Video S2*).

Several bailout techniques have been described to address THV delivery failure. Wire-assisted techniques include pull and push manoeuvres, use of stiffer wire, or placement of a buddy wire in LV or pigtail in NCC, all of which aim to improve co-axiality by straightening the LV-Aortic angle.^9,10^ In the balloon-assisted technique, the balloon is positioned across the AV in either an inflated or partially deflated state, to open the commissure, reduce resistance, and facilitate THV advancement.^9,10^

Snare-assisted technique, primarily described for self-expanding valves, involves pulling the delivery system towards inner curvature of the aorta to improve coaxial alignment.^11,12^ However, the snare placement must be performed before the AV crossing, and therefore, it requires anticipation of difficult THV delivery. In our case snaring was not feasible because the AV had already been predilated and THV was positioned in aorta with stiff wire in the LV.

In our case, repeated THV impingement within the large non-coronary sinus occurred due to delivery along the outer curvature of aorta. We therefore employed a modified shoe-horn technique by fully inflating a large balloon within the non-coronary sinus.

The conventional ‘shoe-horn technique,’ involved balloon placement across the AV with THV advancement during the balloon deflation.^9,13^ In our case, this approach was unsuccessful (see Supplementary material online, *Figure*), necessitating modifications. Unlike previously described balloon nudge technique,^14^ a large balloon was fully inflated within non-coronary sinus, which occupied the space where the valve repeatedly engaged, reduced outer-curvature bias, effectively decreased the functional delivery angle, and provided a low-friction interface that facilitated the valve advancement in a shoe-horning manner (see Supplementary material online, *Figure*).

This case highlights an under-recognized mechanism of THV delivery failure in BAV. The aorta was not horizontal in the traditional sense, as the LV-Aortic angle was 37 degrees, but due to RCC-NCC fusion and calcified raphe, TAVI valve trajectory from the outer curve of aorta towards left sinus, behaved like horizontal anatomy (*Figure 3*, Supplementary material online, *Video S2*). Recognition of this anatomy may allow operators to anticipate delivery challenges and consider alternative methods like, using a balloon-expandable valve with steerable delivery system or upfront snare-assisted technique.

### Limitations

The modified shoe-horn technique was successful in the present case; however, it may not be universally applicable and should be considered as a bailout option in selected anatomical scenarios.

## Conclusions

Pre-procedural CT may not be able to fully predict valve delivery especially in BAV with fusion of NCC and RCC. Fusion of NCC and RCC may behave like horizontal anatomy even with a benign looking LV-Aortic angle. Operators must anticipate difficulty in such cases and be prepared for advanced troubleshooting strategies in case of difficulty delivering THV. This modified shoe-horn balloon assisted technique may serve as effective bailout strategy in this setting.

## Lead author biography


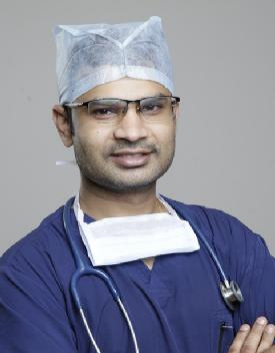


Dr. Nilkanth Patil has obtained his cardiovascular specialization degree (DM Cardiology) at the All India Institute of Medical Sciences (AIIMS) New Delhi. After cardiac specialization he has worked in Delhi and in Ontario, Canada as a fellow in department of Cardiology in London Health Sciences Centre (LHSC) and is now working as senior interventional cardiologist in AIG Hospitals, Gachibowli, Hyderabad. His areas of interest include Coronary and Structural Heart Intervention, cardiac imaging by echocardiography, Cardiac CT and Cardiac MRI

## Supplementary Material

ytag616_Supplementary_Data

## Data Availability

All relevant data are included with the article and its Supplementary files.
